# Medicinal plants and their effects on diabetic wound healing

**DOI:** 10.14202/vetworld.2019.653-663

**Published:** 2019-05-11

**Authors:** Oluwafemi O. Oguntibeju

**Affiliations:** Phytomedicine and Phytochemistry Group, Department of Biomedical Sciences, Faculty of Health and Wellness Sciences, Cape Peninsula University of Technology, Bellville, 7535, South Africa

**Keywords:** Diabetes, healing, infection, medicinal plants, wounds

## Abstract

Wounds have a serious negative impact on the health-care economy of a country, especially on the economy of developing countries where resources are poor and funding is very limited. It is presumed that about 80% of people living in developing countries use traditional medicines which are majorly prepared from medicinal plants to meet their primary health-care needs. Due to the large reservoir of medicinal plants and adequate traditional knowledge on wound healing, many people in Africa and other developing countries use medicinal plants in the treatment of diabetic wounds and related complications. Wound healing in the external and internal biological organs involves a series of complex overlapping processes which demand excellent communication between cells. It is an orderly and highly controlled process characterized by hemostasis, inflammation, proliferation, and remodeling. Diabetes is a global health problem predicted to rise to over 642 million by 2040. The propelling factor responsible for the increase in morbidity and mortality of diabetes is linked to vascular complications as well as the failure of the wound healing processes in diabetic state. Different approaches have been adopted in the treatment of diabetic wounds, and medicinal plants are certainly one of those approaches that have drawn global attention. In this review paper, the effects of medical plants on wound healing in diabetic state as well as factors affecting wound healing and the mechanism of action of medicinal plants are examined.

## Introduction

Diabetes mellitus is a complex disorder resulting from dysregulated glucose sensing or insulin secretion, autoimmune-mediated β-cell destruction in type 1 diabetes or insufficient compensation for peripheral insulin resistance in type 2 diabetes [1-3]. Hyperglycemia, resulting from uncontrolled glucose regulation, is a link between diabetes and diabetic complications [4]. Hyperglycemia and dyslipidemia are two devastating concomitants of diabetes known to play a major role in creating secondary disorders such as macro- and micro-vascular complications [5]. Hyperglycemia plays a key role in increased protein glycosylation [6] while oxidation of glucose produces free radicals that oxidize low-density lipoproteins or favor lipoperoxidation of membrane lipids causing damage to cellular membranes [7-10]. The prevalence of diabetes in the African continent which is known to be prompted by various factors is on the increase. Increase in the morbidity and mortality of diabetes is due to the development of both macro- and micro-vascular complications as well as the failure of the wound healing process [11]. Diabetic wounds are slow to heal, are difficult to manage, and could last for weeks, thereby posing a serious challenge to manage in a clinical setting. The exact pathogenesis of poor wound healing in diabetic wound is not adequately understood. However, human and animal studies show impairment in different phases of the wound healing process [2,8,12-15].

Wound involves a disturbance in the cellular, anatomical, and functional epithelial integrity of the skin consequent to physical, chemical, thermal microbial, or immunological insult; followed by disruption of the structure and function of underlying normal tissue [12,16,17]. The fundamental response in wound healing involves a process of connective tissue repair and is characterized by four overlapping phases such as hemostasis, inflammation, proliferation, and remodeling in which the repair process requires the coordination of different cells, growth factors, and cytokines [18,19]. The mechanisms responsible for delay in wound healing in diabetic patients are not fully understood. Delay in collagen synthesis and impairment in epithelial formation coupled with reduced angiogenesis have been observed during the proliferative phase of the healing process [20-23]. Other factors implicated in the delayed healing process include reduced production of growth factors, vascular endothelial growth factor (VEGF), delayed inflammatory response, excessive protease activity, and impaired nitrite oxide synthesis [24,25].

Approved growth factor and cell therapies for diabetic foot ulcers and other diabetic-related wounds are not usually available during treatment, and improper wound healing control could degenerate into diabetic foot ulcer or possibly into amputation especially in poor resource settings. However, it is of interest to note that nature has created a platform for medicinal agents in the treatment of various ailments and diseases including diabetic wounds. It is important to note that many modern therapeutic agents used in orthodox medicine were derived from medicinal plants. Furthermore, different factors such as bacterial resistance, environmental degradation, and pollution coupled with irrational applications of orthodox medicines have prompted renewed interests in the use of medicinal plants as effective and safer alternatives in the management of various infections such as diabetic wounds [26]. In African and Asian countries, the use of medicinal plants in the treatment of ulcers, boils, sores, and wounds is common knowledge and practice. Report from the clinical study, on the use of two herbal formulae (F1 and F2) in diabetic foot ulcer patients, prevented 85% of legs from limb amputation [27] demonstrating the efficacy and reliability of medicinal plants. In the study of Wong *et al*. [27], the principal component herbs, Radix Astragali and Radix Rehmanniae, were effective in enhancing fibroblast proliferation – the main step in wound healing [28,29]. The presence of bioactive compounds in plants has prompted scientists to investigate the role of medicinal plants to assess their potential wound healing properties and isolate chemicals associated with wound healing. This review paper examines wounds in diabetic condition and the role of medicinal plants in the healing of wounds in diabetic condition.

## Factors Affecting Wound Healing

In general, wound healing is viewed as an interaction between a complex cascade of cellular and biochemical activities culminating in the restoration of structural, functional integrity, and increased strength in injured tissues. The phases of wound healing usually go on in a fashionable and time-dependent manner. Any disruption in the process of wound healing may potentially lead to chronic wound or pathological scarring [30,31]. There are various factors that affect wound healing, and a good understanding of these factors and their possible influence on wound healing may be important in the development of therapeutic agents for wound healing in diabetic and non-diabetic conditions. Factors that affect wound healing are discussed below.

### Wound site

The site of the wound is an important factor in wound healing as wound infection is a common reason for impaired wound healing [2,32]. *Staphylococcus aureus* and *Pseudomonas aeruginosa* are few of the organisms responsible for wound infection, and reports have found that *S. aureus* is the main pathogen associated with diabetic foot infection [33-36].

### Immune state

Various components of the immune system are affected in patients with diabetes. It has been reported that polymorphonuclear leukocyte function is reduced particularly in the presence of acidosis while leukocyte adherence, chemotaxis, and phagocytosis may also be negatively affected in diabetic state [37-40], consequently causing delayed wound healing. Antioxidant systems that participate in bactericidal activity may be impaired in diabetic state, making the wounds in diabetic patients to be susceptible to infection [41,42]. Diabetes is a risk factor for bacteria in patients with pneumococcal pneumonia and is linked to increased mortality [18,43].

### Age

There seems to be a relationship between the ages of an individual and wound healing process [44]. Wound healing seems to be delayed in older age. This is possibly due to the fact that fibroblast growth and activity decrease in older people while collagen synthesis and wound contraction are also reduced in injured older people [18,44].

### Disease state

*S. aureus* and beta-hemolytic streptococci are treated as pathogens in early diabetic foot infections. Studies have reported a higher incidence of bacterial infection in diabetic women than in non-diabetic women [44]. It seems that diabetic patients are more susceptible to wound infection. Greenhalgh [45] reported a higher incidence (11%) in wound infection in diabetic patients than in the general patient population.

### Reactive oxygen species (ROS)

The high concentration of ROS could induce serious tissue damage which could lead to neoplastic transformation, further leading to the impaired healing process by inducing cellular, DNA, proteins, and lipids damages [12,17].

### Diet

Diet has been reported to affect wound healing. It was observed that serum albumin level of 3.5 g/dl or more is required for adequate wound healing. Decreased level of protein could negatively affect collagen synthesis thereby impairing wound healing [12,46].

## Mechanism of Wound Healing

Wound healing is a complex process involving highly regulated steps of biological events, consisting a set of coordinated interactions between cells in the dermis and epidermis. Diabetes is associated with abnormalities in connective tissue, and this contributes to impair wound healing, leading to the formation of chronic ulcer. Briefly, the key four inter-related processes involved in wound healing are described below.

### Hemostatic phase

Following injury, platelets adhere to exposed type 1 collagen and become activated, secreting glycoproteins leading to platelet aggregation. The complex secretes factors that interact with each other to stimulate intrinsic clotting cascade through the production of thrombin; thrombin, in turn, stimulates the formation of fibrin from fibrinogen. The fibrin mesh coupled with platelet, aggregate into a stable hemostatic plug. It is known that within minutes of injury, blood vessels constrict, and reducing the degree of hemorrhage through different steps which allow hemostasis to be achieved [13,31].

### Inflammation phase

There is an overlapping role between the hemostatic phase and the inflammation phase. The inflammatory phase seems to launch the hemostatic mechanisms to urgently stop blood loss from the wound or injury site. This phase may last for up to 2 weeks. The inflammatory phase is marked by vasoconstriction and platelet aggregation to induce blood clotting followed by vasodilation and phagocytosis to produce inflammation at the wound site [47].

### Proliferative phase

This phase which follows the inflammatory phase, lasting from 2 days to 3 weeks, consists of key steps which include granulation, contraction, and epithelialization. During granulation, fibroblasts form a bed of collagen with the production of new capillaries. In the past two steps, wound edges pull together to reduce the defects (contraction) while fresh epithelial and scar tissues are formed over the wound site (epithelialization) [30,31].

### Remodeling phase

During this phase, new collagen is synthesized, accompanied by increased tissue tensile strength due to intermolecular cross-linking of collagen through Vitamin C dependent hydroxylation. It is believed that this phase lasts from 3 weeks to 2 years [48].

## The Role of Medicinal Plants in Wound Healing in Diabetic Model

As a consequence of ethnobotanical survey, many species of plants and herbs with wound healing activities have been identified in Africa and other developing countries. The use of medicinal plants in wound management and care involves disinfection, debridement, and the provision of adequate environment for natural healing process [49]. It is assumed that ingredients from medicinal plants are less toxic and have fewer side effects compared with orthodox therapeutic agents; hence, the increased and renewed interest in the use and application of medicinal plants in the wound healing process both in diabetic and non-diabetic conditions. Impairment in the healing of diabetic wound is seen as a serious health challenge for health professionals globally, and this is linked to the non-specific etiology in some cases; therefore, one of the therapeutic approaches for treatment is the application of medicinal plants particularly in poor resource settings [8].

### 

#### Rosmarinus officinalis

**Figure-1:**
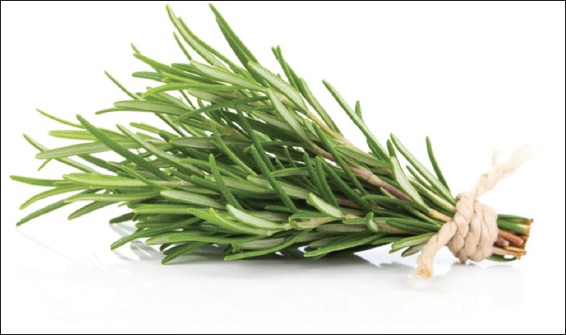
*Rosmarinus officinalis*. Source: www.naturalfoodseries.com.

**Figure-2:**
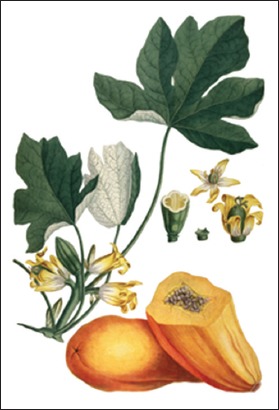
*Carica papaya*. Source: www.pfaf.org.

**Figure-3:**
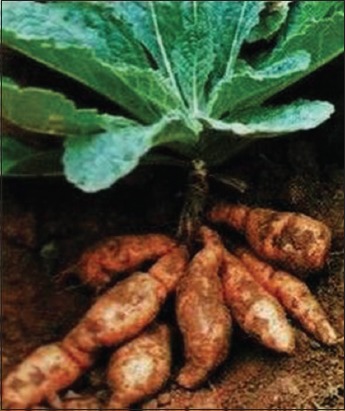
Radix Rehmanniae. Source: www.tradeindia.com.

**Figure-4:**
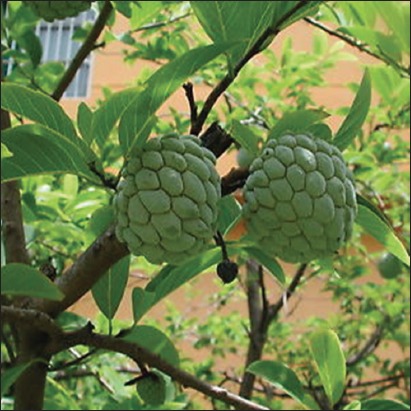
*Annona* *squamosa*. Source: www.ebay.com.

**Figure-5:**
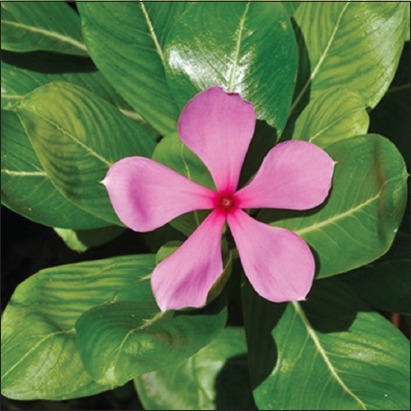
*Catharanthus* *roseus*. Source: www.invasives.org.za.

**Figure-6:**
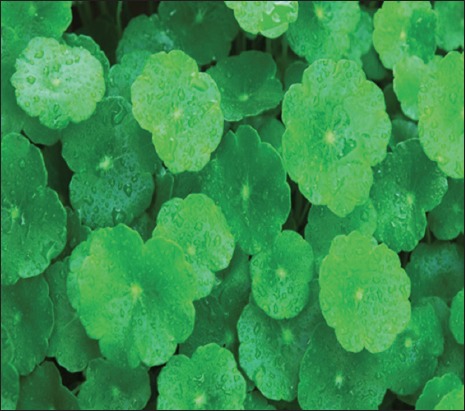
*Centella* *asiatica*. Source: www.lipotherapea.com.

**Figure-7:**
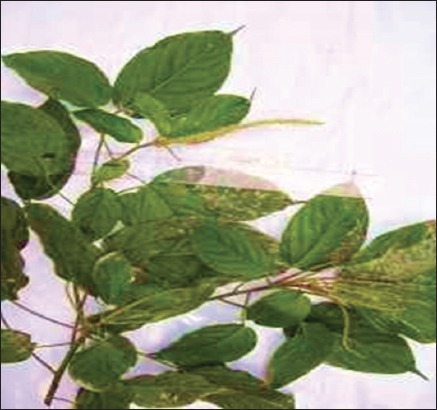
*Acalypha* *langiana*. Source: www.intermountainbiota.org.

**Figure-8:**
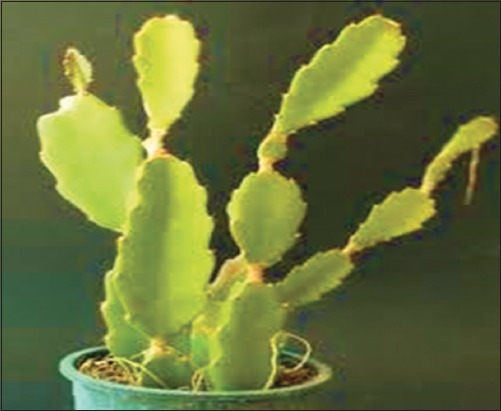
*Hylocereus* *undatus*. Source: www.cactusgarden it.

**Figure-9:**
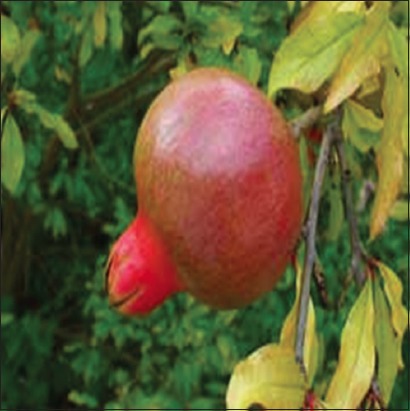
*Punica granatum*. Source: www.pfaf.org.

**Figure-10:**
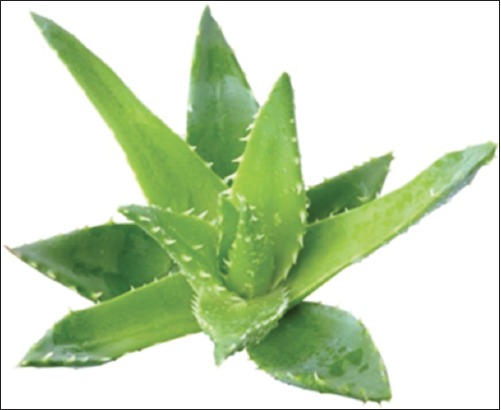
*Aloe* *vera*. Source: www.duduosum.com.

**Figure-11:**
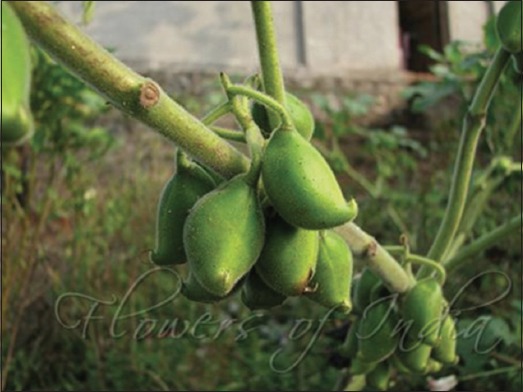
*Martynia* *annua*. Source: www.flowersofindia.net.

This plant is known for its antioxidant and antibacterial activities. In the animal model, Abul-Al-Basal [50] examined the healing potential of the extract of the plant on full-thickness excision cutaneous wounds in alloxan-induced diabetic BALB/c mice. For this study, male BALB/c mice of 6 weeks old weighing 18-20 g were used. Diabetes in the mice was induced by injecting 0.2 ml of alloxan monohydrate intraperitoneally. Only animals that were diabetic after injection of alloxan were used in the experiment. Following the experiment, various tests were performed including wound contraction, granulation tissue, and histology. Results showed that on day 3, the wet weight of granulation tissue has significantly increased compared to the untreated controls. Quantitative measurements of wound size were routinely used to assess initial wound size before and after debridement as well as progress toward wound closure. The wound contraction rate was measured as the percentage reduction in wound size on days 3, 6, 10, and 15 following wounding. Significant progress in the percentage of wound contraction was observed in the group treated with the extract compared with the untreated. Histological examination showed that the enhancement of the healing process was observed on days 6 and 15 after wounding in the treated group. The author concluded that the essential oil from the aerial parts of the plant demonstrated superior significant healing effect over the aqueous extract when topically applied on the wound of diabetic mice and that the healing property of the plant could be linked to the powerful antimicrobial, anti-inflammatory, and antioxidant activities of the plant [49,51]. The medicinal plants and their names are shown in Figures 1-11.

#### Carica papaya

The extract of *C. papaya* has been reported to display antimicrobial, antioxidant, and anti-inflammatory activities [52]. To investigate the wound healing activity of *C. papaya* in a diabetic animal model, Nayak *et al*. [53] collected unripe fruits of *C. papaya* locally and prepared it according to the previously described method [54]. Following the induction of diabetes, rats were inflicted with excision wounds according to the method of Morton and Malone [55]. Animals were anesthetized with 1 ml of intravenous ketamine hydrochloride and shaved on both sides of the back with an electric clipper. A full thickness excision wound of the circular area was created along a marked area. The wound was left open and animals were observed for any infection. Animals that showed signs of infection were excluded from the study and replaced. Animals were divided into five groups (Group 1-5) with 6 animals per group. The animal in Group 4 (diabetic experimental group) was treated topically with the extract of *C. papaya* and wounds measured on days 1, 5, and 11 for all groups. The authors reported that a significant increase in the wound healing activity was observed in rats treated with fruit extract. In the excision wound model, animals of Groups 2 and 4 showed a decrease in the epithelialization period and increased the percentage of wound contraction when compared with the animals in Groups 1, 3, and 5. In the dead space wound model, the extract-treated animals in Groups 2 and 4 showed significantly higher levels of hydroxyproline compared with animals in the normal and diabetic control groups. A significant increase was equally noted in the dry and wet weight of the granulation tissue in the animals treated with the extract. In animals that did not receive the extract, the wound was hard and crusty with undermined margins and generally unclean. It is known that wound healing deficits in diabetes are varied and inter-related and the defect is associated with impaired blood flow and oxygen release from increased blood sugar, decreased collagen and fibronectin synthesis from protein malnutrition, impaired local immune and cell defenses, and reduced anabolic activity with reduced insulin and growth hormone and that hyperglycemia affects neutrophil function which includes migration, chemotaxis, adherence, and phagocytic and bactericidal activity [56]. The wound healing effect of *C. papaya* is related to its proteolytic enzymatic activity and antimicrobial activity which are found in chymopapain and papain-major constituents of *C. papaya*. The absence of biofilm observed in diabetic animals treated with the extract shows that enzyme constituents of *C. papaya* were able to breakdown the biofilm defenses which protects against UV light and oxygenation and promoted bacterial imbalance. It is believed that the papain in *C. papaya* provides enzymatic debridement of wounds apart from being rich in Vitamin C which is important in the conversion of proline to hydroxyproline [54].

### Radix Rehmanniae

The role of Radix Rehmanniae in the healing of foot ulcers in traditional Chinese medicine has been described [28]. To investigate the wound healing effects of Radix Rehmanniae in an animal model of diabetic foot ulcer, Lau *et al*. [28], used female albino Wistar rats which are commonly used in ulcer and diabetic studies and female Sprague Dawley rats used in inflammation studies. In this study, the induction of diabetes and foot ulcer was done according to the previous method [28]. The wound healing effect of Radix Rehmanniae was compared with the group that received water on days 4, 8, 13, and 18 in the diabetic rat foot ulcer model. A significant reduction of the ulcer area from days 8 to 18 was observed in the group that received Radix Rehmanniae extract. With respect to tissue regeneration, the epithelialization and scar formation were not well developed in a group that was given water while they were well developed in the group that received treatment with Radix Rehmanniae extract. It is important to note that rats that received Radix Rehmanniae extract had significant expression of VEGF starting from day 4 and lasted till day 13. Diabetes has been reported to inhibit angiogenesis [57]. Radix Rehmanniae demonstrated its effectiveness in promoting healing of diabetic foot ulcer healing in rats through the mechanism of tissue regeneration, angiogenesis, and inflammation control.

#### Annona squamosa

*A. squamosa* is known as custard apple. It is cultivated in India where local people use the leaves and seeds for the treatment of diabetes and other diseases such as fever and ulcer [58]. Ponrasu and Suguna [59] assessed the efficacy of the ethanolic leaf extract of the plant on wound healing in streptozotocin (STZ)-induced diabetic rats. For the experiment, diabetes was induced in healthy male Wistar rats by a single intraperitoneal injection of STZ (50 mg/kg body weight). After induction of diabetic, rats were shaved from the back and a 2 cm^2^ full thickness open excision wound was made by removing a patch of the skin using anesthesia [60]. The control rats remained untreated while the treated rats were administered with 200 ml (100 mg/kg body weight) of the extract topically once daily until the wounds were healed. Results showed that wounds treated with the extract contracted completely on day 17 compared to control groups which showed 85% of contraction. There was also a reduction in wound size, improved tensile strength, and epithelialization. The macroscopic analysis of the wounds revealed that the groups treated with the extract required a total period of 17 (non-diabetic group) and 22 days (diabetic) for complete healing. Diabetis mellitus is associated with different changes in connective tissue metabolism which makes diabetic patients to experience delayed wound healing. However, as can be seen from this report, treatment with *A. squamosa* was found to significantly enhanced both excision and incision wounds and its wound healing activity has been said to be related to its high phenolic content by enhancing collagen synthesis and wound contraction [31,59].

#### Catharanthus roseus

*C. roseus* (also known as *Vinca rosea*) is a shrub with distinct purple or white flowers that have its origin in Madagascar. The hypoglycemic nature of the plants has been linked with the presence of various phytochemicals distributed throughout the plant. *C. roseus* was reported by Rasinenis *et al*. [61] to be an antihyperglycemic plant rather than a hypoglycemic plant as it increases glycolysis and glucose oxidation (through the Shunt pathway). In a study Rasinenis *et al*. [61] performed in diabetic rats, a 77.7% blood glucose reduction in treated diabetic rats was noted after 60 days of treatment. To validate the earlier findings, Singh *et al*. [62] observed an increase in the activity of glucose metabolic enzymes as well as a decrease in lipid peroxidation a driving factor in the development of diabetic complications.

The influence of the ethanol extract of *C. roseus* on wound healing in diabetic rats was performed by Nayak [15]. The motivation for the study was based on the fact that current approaches for treating chronic diabetic wounds such as debridement, irrigation, antibiotics, tissue grafts, proteolytic enzymes, and corticosteroids are believed to have side effects. The leaves of *C. roseus* (100 g) were washed with water, dried, and the leaves ground into solution using 200 ml of ethanol. The contents were filtered and the clear solution used for the study. Healthy female Sprague Dawley rats weighing 180-200 g were used. Animals were divided into five groups. Animals in Groups 3, 4, and 5 were induced with diabetes using STZ (50 mg/kg body weight) in a single dose intraperitoneally. All the rats were anesthetized with 1 ml of intravenous ketamine hydrochloride (10 mg/body weight) and shaved on both sides of the back and the area of the wound to be created was marked on the back of the animals with methylene blue. The full thickness of 2.5 cm length and 0.2 depth of the excision wound were created along with the markings with toothed forceps, surgical blade, and pointed scissors. Groups 2 and 4 were treated topically with the extract, applied as a single layer thickness to the wound for 10 days. Postoperatively, granulation tissue formed was excised, and wet weight was recorded. Results showed that topical application of the extract to the wound of diabetic experimental rats (Group 4) significantly increased the rate of wound contraction when compared with the diabetic control and normal control (Groups 3 and 1). Granulation tissue had proliferated, showing a significant increase in dry weight in the diabetic experimental group compared with animals in the standard test group. Furthermore, animals that received the extract showed fast lay down of collagen. Animals that were not treated with *C. roseus* extract show wounds that appear hard and crusty. It is presumed that constituents such as alkaloids, triterpenoids, and tannins of *C. roseus* may have played a major part in the process of wound healing in diabetic rats due to their astringent and antimicrobial activities which may be responsible for wound contraction and increase rate of epithelialization [15].

#### Centella asiatica

*C. asiatic*a, is commonly used as a medicinal herb in Ayurvedic medicine, traditional African medicine, and traditional Chinese medicine. It has been known to promote fibroblasts proliferation and collagen synthesis [63]. Nganlasom *et al*. [64] made excision wounds on day 3 after induction of diabetes. The animals were cut on the left side of dorsal flank skin in the experimental rats. Excision wounds were made following anesthesia by cutting out a 15 mm by 15 mm piece of skin from the shaved area. The study concluded that *C. asiatica* could facilitate wound healing under diabetic condition in the animal; however, additional studies are suggested to identify specific ingredients that are responsible for the healing effects.

#### Acalypha langiana

*A. langiana* is a herbal plant that grows in the wild and the leaves of the plant has been used in traditional medicine to treat wounds and bacterial infections [25]. For this study, the authors made use of the aqueous extract of fresh leaves of *A. langiana*. The rats were made diabetic by a single injection of STZ. Wounds were made on rats that showed high blood glucose (>250 mg/dl) on day 7 following induction of diabetes and two types of wounds were made (excision and incision wounds) in experimental rats. Topical application of the aqueous leaves extract of *A. langiana* demonstrated a significant and dose-dependent effect on the healing process on the diabetic rats. Specifically, incision wounds treated with the extract showed a significant increase in tensile strength. Results also indicate that tissue regeneration was much faster in granulation tissue sections in the extract treated group compared to the control wounds.

#### Hylocereus undatus

This plant is widely distributed in Brazil with large fragrant flowers that open only in the night and are believed to play an important role in food and traditional medicine. Specifically, its leaves and flowers are used as a hypoglycemic agent [65,66]. To assess the wound healing properties of the plant [67], used the leaves, flowers, and fruits extracts in the experiment. Wistar rats with weight 170-200 g (male and female) were used. The rats were induced with diabetes by given a single injection of STZ (50 mg/body weight) intraperitoneally. One excision wound was inflicted by cutting away 500 mm full-thickness skin from a predetermined area on the back of each rat. Topical applications of the extract of the plant revealed a significant increase in the healing process in the diabetic rats. In the diabetic rats, wounds treated with 0.5% solutions of the flowers and leaves extract showed a significant increase in the tensile strength compared with the control. Wounds treated with the flower extract also showed a significant increase in the collagen content of the granulation tissues compared to the untreated control. The study confirmed that topical application of the aqueous extracts of both leaves and flowers yielded significant wound healing activity.

#### Punica granatum

*P. granatum* is an important medicinal plant in the Middle East, and the flowers are used as antibacterial, antifungal, and antiviral agents and widely used in the treatment of wounds [68]. In experimental work, Pirbalouti *et al*. [68] investigated the wound healing effects of the plant using male Wistar rats. After 15 h of fasting, the animals were injected intraperitoneally with 125 mg/kg body weight of alloxan monohydrate. Following the determination of glucose to ascertain the diabetic state of the animals, wounds were made on the rats with glucose level >250 mg/dl. Animals were divided into the following groups in which Group 1 constituted of normal rats treated with ointment base (control); Group 2 – diabetic group treated with ointment base (control); and Groups 3 and 4 treated with ointment base containing extracts (0.2%) while the last group received standard drug (nitrofurazone). Results indicate that on day 9, tissue regeneration was greater in the skin wound treated with ointment base containing the extracts. The wound healing effects of *P. granatum* in diabetic animal were evident on day 18 of treatment, and the result possibly justifies the use of *P. granatum* in wound treatment in traditional medicine.

#### Aloe vera

*A. vera* has been recognized and used in traditional medicine in different cultures across the globe in the treatment of various disease conditions. It has been shown to possess antidiabetic, anti-inflammatory, and antibacterial activities [8,69]. Male Wistar rats were used in an *in vivo* study to evaluate the wound healing activity of the plant extract. After the induction of diabetes using STZ, wounds were created on day 7. Two types of wounds were created: Excision and incision wounds and the wounds were treated with extract of *A. vera* for specific days. To assess the rate of wound contraction, excisions were traced on a transparent paper with a millimeter scale, and the change in wound size was calculated as the percentage wound area that had healed. The period of epithelialization of the wound was indicated as number of days taken for complete epithelialization. There was a steep increase in collagen content after day 4 of granulation tissues. There was also increase in protein and DNA contents of the granulation tissues in animals treated with plant extract. In conclusion, the study shows that *A. vera* extract increased wound healing in diabetic state and suggests that treatment with *A. vera* may have beneficial effects on the various phases of wound healing such as fibroplasia, collagen formation, and contraction which culminated in faster healing compared to untreated animal [69].

#### Martynia annua

This is a glandular hairy annual herb used predominantly in the treatment of epilepsy and tuberculosis. It is also used for the treatment of sore throat, inflammation, and wounds [70,71]. The leaves of the plant were collected, identified, and processed according to the previously described method [72]. An excision wound was made in Wistar rats after induction of diabetes through injection with STZ. Wound treated with the extract was observed to show significant contraction in wound compared to those of the control group. The hydroxyproline content in animals treated with plant extract was also found to be significantly higher than that of control. Histological examination showed well-organized collagen fibers, increase in fibroblast cells in animals treated with extract of *M. annua* [73].

## Other Plants use in Wound Treatment (Non-diabetic Wounds)

There are several other plants that are used traditionally in the treatment of wounds in Africa and Asia. However, in this review, the focus is on medicinal plants specifically used in the treatment of wounds in diabetic condition. Other plants that are generally used in the treatment of wounds include *Morinda* *citrifolia*, *Lycopodium serratum*, *Cecropia peltata*, *Lawson alba*, *Ginkgo* *biloba*, and *Moringa* *oleifera* [49]. Plants such as *Hibiscus* *sabdariffa*, *Capsicum* *frutescens*, *Ageratum conyzoides*, *Aframomum* *melegueta*, *Ocimum* *gratissimum*, *Euphorbia heterophylla*, *Sida acuta*, *Parkia biglobosa*, *Vernonia* *amygdalina*, and *Tridax* *procumbens* have also been reported to demonstrate wound healing effects [74]. Table-1 shows various medicinal plants examined in this review and their biological activities.

**Table-1:** Medicinal plants and their effects in diabetic would healing.

Medicinal plants	Effects/Activities
*Rosmarinus officinalis*	Antioxidant, antibacterial, and inflammatory effects
*Carica papaya*	Proteolytic enzymatic and antimicrobial activities
Radix Rehmanniae	Tissue regeneration, angiogenesis, and anti-inflammatory activity
*Annona squamosa*	High phenolic content, enhancing collagen synthesis, and contraction
*Catharanthus roseus*	Astringent and antimicrobial activities leading to increased rate of epithelialization
*Centella asiatica*	Antioxidant and anti-inflammatory activities
*Acalypha langiana*	Increased tissue regeneration
*Hylocereus undatus*	Increased tensile strength
*Punica granatum*	Antibacterial and antifungal effects
*Aloe vera*	Antidiabetic, anti-inflammatory, and antibacterial activities
*Martynia annua*	Antibacterial and antioxidant activities

## Mechanism of Action of Medical Plants on Wound Healing

The increasing demand and availability of medicinal products have prompted the need to isolate and understand the principles responsible for their therapeutic activities and effectiveness. For instance, stimulation of fibroblasts by plant extracts has been observed as one of the mechanisms by which medicinal plants enhance the wound healing process [60]. It is reported that certain ingredients contained in medicinal plants possess growth factor-like activity or has the ability to stimulate an early expression of growth factors [75,76]. Extracts of medicinal plants have been documented to arrest bleeding from fresh wounds, inhibits microbial growth, and promotes wound healing [28]. In addition, the effects of medicinal plants on wound healing may be linked to the free radical scavenging action of compounds in the extracts acting either singly or synergistically. These active compounds enhance the process of wound healing by increasing the viability of collagen fibrils, by increasing the strength of collagen fibers, increasing the circulation or prevention of cell damage, or promoting DNA synthesis [48,77,78]. ROS at a high level can induce cellular damage which could constitute a barrier to the healing process by damaging the DNA, proteins, and lipids. It is believed that extracts of medicinal plants with antioxidant activity could be helpful therapeutic agent in promoting wound healing. Ingredients such as triterpenes, alkaloids, flavonoids, tannins, and saponins are believed to play key roles in wound healing effects of medicinal plants [17]. The report shows that flavonoids possess potent antioxidant and free scavenging and antioxidant activity which are known to reduce lipid peroxidation, which, in turn, reduce cell necrosis and improved vascularity. Tannins act as a free radical scavenging agent while triterpenoids and saponins enhance wound healing by antioxidant and antimicrobial activity and are believed to be promoting wound contraction and increased epithelialization [17,79,80]. Some of the medicinal plants also contain trace elements such as zinc and vitamins such as Vitamin C. For instance, zinc act as cofactor in many enzymatic reactions including zinc-dependent matrix metalloproteinases which enhance keratinocyte migration during wound repair. On the other hand, Vitamin C contributes to wound healing as an important ingredient in collagen formation [81].

## Further Research

Further research is needed to isolate, identify, and purify active ingredients in the plant extracts that are involved in wound healing processes in diabetic and non-diabetic conditions. Application of plant extracts as a possible adjuvant in the orthodox treatment of wounds should be scientifically explored. Large clinical trial on the use of medicinal plants in wound healing in diabetic and non-diabetic individuals should be conducted. Therapeutic application of cytokines, growth factors and their soluble receptors could be studied to determine the extent of their involvement and acceptability in wound healing and treatment. Fibrotic processes are continuous and characterized by collagen synthesis, downregulation of degradative enzymes involved in removing scar tissue and fibrosis has been reported to be inhibited by antibodies, peptide receptor antagonists. Research into interactions between fibrotic processes and antibodies could also provide useful information on wound healing. A better understanding of the mechanisms of initiation, progression and resolution of wound healing could lead to the discovery of new therapies. Despite limitations on the degree and extent of the applications of medicinal plants in the treatment of diabetic wounds, it shows considerable promise and can indeed herald exciting new therapeutic strategies in wound healing.

## Conclusion

Wound healing activities of medicinal plants in diabetic condition have recorded some appreciable efficacy as reported in this paper. Despite the limitations in terms of clinical trials, the majority of people especially in developing countries continue to depend on medicinal plants in the treatment of various diseases and infections including diabetic wounds. In one particular case, the application of ointment derived from medicinal plants prevented 85% of infected diabetic wound from the amputation of the legs. Further research and clinical trials are recommended to confirm the efficacy and safety of specific medicinal plants and their mechanisms of action on diabetic wound healing.

## Author’s Contributions

OOO conceptualized the research idea, conducted the literature search, wrote and revised the manuscript.
